# Encapsulation of Functional Plant Oil by Spray Drying: Physicochemical Characterization and Enhanced Anti-Colitis Activity

**DOI:** 10.3390/foods11192993

**Published:** 2022-09-26

**Authors:** Hao Zhang, Zhenxia Xu, Zhixian Qiao, Xu Wang, Hu Tang, Chen Yang, Fenghong Huang

**Affiliations:** 1 Oil Crops Research Institute of the Chinese Academy of Agricultural Sciences, Oil Crops and Lipids Process Technology National & Local Joint Engineering Laboratory, Key Laboratory of Oilseeds Processing of Ministry of Agriculture and Rural Affairs, Hubei Key Laboratory of Lipid Chemistry and Nutrition, Wuhan 430062, China; 2 Institute of Hydrobiology, Chinese Academy of Sciences, Wuhan 430072, China; 3 Hubei Collaborative Innovation Center for Animal Nutrition and Feed Safety, Huazhong Agricultural University, Wuhan 430070, China; 4 Institute of Food & Nutrition Science and Technology, Shandong Academy of Agricultural Science, Jinan 250100, China

**Keywords:** flaxseed oil, encapsulation, oil powder, colitis, gut microbiota

## Abstract

In this study, an encapsulation system was developed for functional plant oil delivery. Through a series of orthogonal experiments and single factor experiments, the raw material compositions, emulsification conditions, and spray drying conditions for the preparation of flaxseed oil and safflower seed oil powders were optimized, and the final encapsulation efficiency was as high as 99% with approximately 50% oil loading. The storage stability experiments showed that oil powder’s stability could maintain its physicochemical properties over six months. Oral supplementation of the spray-dried flaxseed oil powder exhibited a significant and better effect than flaxseed oil on alleviating colitis in C57BL/6J mice. It suppressed the pro-inflammatory cell factors, including IL-6 and TNF-α, and repaired gut microbial dysbiosis by increasing the microbial diversity and promoting the proliferation of probiotic taxa such as *Allobaculum*. This work suggests that spray-dried flaxseed oil powder has great potential as a nutraceutical food, with spray drying being a good alternative technique to improve its bioactivity.

## 1. Introduction

Plant-based functional oil, such as flaxseed oil and safflower seed oil, contains a rich source of polyunsaturated fatty acids (PUFAs), which have been proven beneficial to human health when consumed in the diet. Previous research has shown that PUFAs can decrease many disease risks (such as coronary heart disease, high blood pressure, and Alzheimer’s disease) and improve mental health and brain function [1,2,3,4]. However, the unsaturated fatty acids within these oil types are prone to oxidation which may result in the formation of harmful products and have disadvantages such as low availability and unpleasant odor, which limit their applications [5].

Transforming functional liquid oil into solid oil powders has become a very attractive process in the food and pharmaceutical industries, as this produces encapsulated products that can provide a physical barrier between the oil and the external environment that can avoid deterioration and unpleasant odor. In addition, the obtained powder forms are more acceptable in the market due to their improved odor and extended shelf-life [6,7].

Previous studies have shown a lot of encapsulation combinations and methods for oil encapsulation, including coacervation, ionic gelation, freeze drying, cross-linking etc., but most of them only focus on the physicochemical properties of the encapsulated products. Limited information exists relating to the confirmation of the health effects of these products [8,9]. The experimental induction of colitis has been used not only to study gut inflammatory processes but also to evaluate the effects on intestinal barrier integrity and homeostasis for various encapsulated products. As representative plant-based oil resources rich in PUFAs, flaxseed oil and safflower seed oil have been widely explored due to their high levels of n-3 PUFA-α-linolenic acid (50–60%) and n-6 PUFA-linoleic acid (>70%), respectively [3,10]. Investigation of the encapsulation of these two oils, as well as the evaluation of the physiochemical properties and the health benefits of the obtained oil powders, can help us learn more about the advantages of encapsulation and contribute to the development of relative nutraceuticals including n-3 PUFA, n-6 PUFA, or their blends.

This study systematically studied the fabrication of an encapsulation system for flaxseed oil and safflower seed oil. Modified starch and maltooligosaccharide were used as wall material combinations, and orthogonal experiments and single factor experiments were designed to obtain the optimal raw material compositions, emulsification, and spray drying conditions. Additionally, the physicochemical properties of these powdered oil products were evaluated. Furthermore, the potential effect of flaxseed oil powder on maintaining the intestinal epithelial barrier in dextran sodium sulfate (DSS)-induced colitis mice were examined. The research will provide further insight into the method of fabricating spray-dried oil powders and their beneficial roles in alleviating colitis.

## 2. Materials and Methods

### 2.1. Materials

Flaxseed oil was purchased from Hongjingyuan Oil Co. Ltd. (Xilingol, China). Safflower seed oil was purchased from Zhongliang Food Marketing Co., Ltd. (Alashankou, China). Fatty acid compositions of flaxseed oil and safflower seed oil are shown in Table S1. Modified starch (HI-CAP 100) was purchased from Ingredion Incorporated (New York, USA). Maltooligosaccharide with a dextrose equivalent (DE) of 18–28 was purchased from Baolingbao Biotechnology Co., Ltd. (Dezhou, China). Mono- and diglycerides of fatty acids were purchased from Jia Li Shi Additives (Hai An) Co., LTD (Haian, China). Petroleum ether with bp range from 30 to 60 °C was used in this study. Other reagents were of analytical grade and were obtained from Sinopharm Chemical Reagent Co. Ltd. (Shanghai, China).

### 2.2. Preparation of Emulsion

The oil emulsion was composed of an aqueous phase and an oil phase. The aqueous phase was prepared by dissolving modified starch and maltooligosaccharide in distilled water, and the oil phase was obtained by dispersing mono- and diglycerides of fatty acids in the oil. A coarse emulsion was then prepared by gradually pouring the oil phase into the aqueous phase with continuous shearing at 11,000 rpm for 15 min with a high-shear mixer IKA-T25 (IKA Instruments Ltd., Staufen, Germany). The secondary emulsion was finally obtained by passing this coarse emulsion through a high-pressure homogenizer ATS AH-Basic (ATS Industrial Co. Ltd., Toronto, Canada) at designed homogenization pressure and homogenization cycles.

### 2.3. Particle Size Distribution of Emulsion

The particle size distribution of emulsion was measured by a dynamic light scattering instrument Mastersizer 2000 (Malvern Instruments, Malvern, UK) at 25 °C. D [0.1], D [0.5], and D [0.9] represent 10%, 50%, and 90% below their sizes. D [3,2] is the surface weighted mean diameter, and D [4,3] is the volume-weighted mean diameter. Span value was obtained by dividing the difference of D [0.1] and D [0.9] by D [0.5] [11].

### 2.4. Preparation of Oil Powders

To prepare oil powders, the emulsion was spray-dried in a QZR-5 spray drier (Linzhou Spray Dryer Co., Wuxi, China). The emulsion was fed into the main chamber through a peristaltic pump with adjustable feeding speed (LongerPump BT100-2J).

### 2.5. Orthogonal Experiment Design

In this study, various types of factors: including raw material compositions, emulsification conditions, and spray drying conditions, can affect the characteristics of the final products when transforming the liquid oil into powdered oil by spray drying. To obtain the products that have targeted total oil content (50%) with high encapsulation efficiency, these conditions were optimized. Based on previous research and our preliminary experiments, the effects of raw materials (including wall ratio, core/wall ratio, solid concentration, and emulsifier content) were firstly evaluated to determine the optimal raw material compositions. Flaxseed oil was used in this section, and the selected raw material factors and respective levels are shown in Table 1. These four different factors were optimized using the orthogonal *L_16_ (4) ^4^* experiment. A total of 16 experimental trials were formulated (Table 2) using the same process parameters: homogenization pressure (20 MPa), homogenization cycles (2 cycles), and inlet air temperature (170 °C).

### 2.6. Characterization of the Oil Powders

#### 2.6.1. Total Oil Content

The total oil content was measured according to an acid hydrolysis method described in the Chinese National Standard GB 5009.6—2016 [12]. Briefly, 2–3 g of powder samples were accurately weighed (precise to 0.0001 g) and added to a test tube. Then, 8 mL of water were added and well mixed, followed by the addition of 10 mL of hydrochloric acid. The test tube flask was then placed in a water bath at 70 ± 5 °C for 40–50 min, during which it was stirred with a glass rod every 5–10 min. The test tube was then taken out and followed by the addition of 10 mL alcohol. The mixture was transferred into a 100 mL mixing cylinder with a stopper; 25 mL of ethyl ether was added several times to wash the test tube, and then the mixture was transferred into the mixing cylinder. After closing the stopper, the mixing cylinder was shaken for 1 min, carefully opening the stopper to release the gas and closing it again to stand for 12 min. The stopper was then opened, and the oil attached to the bottleneck of the mixing cylinder was washed with ethyl ether. Keeping the cylinder standing for 20 min until clear liquid was observed in the upper layer, the supernatant was removed and placed in a weighing dish (previously dried in an oven at 105 °C for 1 h). Another 5 mL of ethyl ether were added to the mixing cylinder to repeat the process. The solvents in the weighing dish were then evaporated, and the dish was dried to constant weight in an oven at 105 °C. The total oil content was calculated according to the following Equation (1):

(1) $$\mathrm{Total}\;\mathrm{oil}\;=\frac{m_{1}-m_{0}}{m}\times 100\%,$$

where *m*_1_ is the weight of the weighing dish and oil, *m*_0_ is the weight of the weighing dish, and *m* is the weight of the powdered samples.

#### 2.6.2. Surface Oil Content

The surface oil content of the encapsulated powders was measured according to the method described in the Chinese Aquaculture Industry Standard SC/T 3505—2006 with a minor modification [13]. In total, 5.0 g of powdered samples were weighed and added to a 250 mL volumetric flask. Then, 30 mL of petroleum ether (bp 30–60 °C) were added, and the flask was slightly shaken by hand for 10 s. The suspension was filtered into a weighing dish which was pre-dried in an oven at 105 °C. The powder residue was washed using 20 mL of petroleum ether, then shaken and filtered into the same weighing dish. This process was repeated twice. The weighing dish was then placed in a water bath at 60 °C to evaporate the organic solvent and dried at 105 °C until it reached a constant weight. The surface oil content was calculated using the following Equation (2):

(2) $$\mathrm{Surface}\;\mathrm{oil}=\frac{W_{2}-W_{1}}{W}\times 100\%,$$

where *W*_1_ is the weight of the weighing dish, *W*_2_ is the weight of the weighing dish and oil, and *W* is the weight of powdered samples.

#### 2.6.3. Encapsulation Efficiency

The encapsulation efficiency was calculated according to the following Equation (3):

(3) $$\mathrm{Encapsulation}\;\mathrm{efficiency}=\frac{\mathrm{Total}\;\mathrm{oil}-\mathrm{Surface}\;\mathrm{oil}}{\mathrm{Total}\;\mathrm{oil}}\times 100\%.$$

#### 2.6.4. Moisture Content

The moisture content of the spray-dried powders was measured according to the hypobaric drying method described in the Chinese National Standard GB/T 5009.3—2016 [14]. In total, 2~10 g powders were added into a weighing bottle with constant weight and weighed (precision to 0.0001 g). The samples were then dried at 60 ± 5 °C under a vacuum drying oven with a pressure of ~45 kPa. The moisture content was calculated via Equation (4):

(4) $$\mathrm{Moisture}\;\mathrm{content}=\frac{m_{2}-m_{3}}{m_{2}-m_{4}}\times 100\%,$$

where *m*_2_ is the weight of the weighing bottle and powdered samples before drying, *m*_3_ is the weight of the weighing bottle and powdered samples after drying, and *m*_4_ is the weight of the weighing bottle.

#### 2.6.5. Acidity Value Analysis

The acidity value was determined according to the cold solvent indicator titration method described in the Chinese National Standard GB/T 5009.229—2016 [15]. The encapsulated oil was extracted from the powders prior to the test. The extracted oil samples were weighed and added to a 250 mL conical flask. In total, 50 mL of ether-isopropanol (1:1) solvent mixture were added to the flask, followed by the addition of three drops of phenolphthalein indicator solution. The mixture was then titrated with 0.1 M potassium hydroxide standard solution. Blank tests were performed without the addition of extracted oil. The acidity value was calculated according to the following Equation (5):

(5) $$\mathrm{Acidity}\;\mathrm{value}=\frac{\left(V-V_{0}\right)\times c\times 56.1\;}{m}\times 100\%,$$

where *V* and *V*_0_ is the consumption of potassium hydroxide standard solution in the main test and in the blank test (mL), respectively, *c* refers to the molar concentration (molarity) of the potassium hydroxide standard solution (mol/L), 56.1 is the molar mass of potassium hydroxide (g/mol), and *m* is the weight of the extracted oil.

#### 2.6.6. Peroxide Value Analysis

The peroxide value was measured with a titration method described in the Chinese National Standard GB/T 5009.227—2016 [16]. The encapsulated oil was extracted from the powders prior to the test. The extracted oil samples were weighed (precision to 0.0001 g) and added to a 250 mL iodine flask. In total, 30 mL of glacial acetic acid-chloroform (3:2) solvent mixture were added into the flask, shaken slightly for 0.5 min, and placed under darkness for 3 min. Then, 100 mL of deionized water were added and shaken. The mixture was then titrated with sodium thiosulfate solution. The peroxide value was calculated according to the following Equation (6):

(6) $$\mathrm{Peroxide}\;\mathrm{value}=\frac{\left(V-V_{0}\right)\times c\times 0.1269\;}{m}\times 100\%,$$

where *V* and *V*_0_ are the consumption of sodium thiosulfate solution in the main test and the blank test (mL), respectively, *c* is the molar concentration (molarity) of the sodium thiosulfate solution (mol/L), 0.1269 is the mass (g) of iodine titrated with 1 mL sodium thiosulfate solution (1 mol/L), and *m* is the weight of the extracted oil.

#### 2.6.7. Powder Morphology

The morphologies of the powdered samples were observed using scanning electron microscopy (SEM, TESCAN Vega3). The samples were coated with gold prior to the tests. The particle sizes of the powdered samples were obtained by analyzing the SEM images with ImageJ software.

### 2.7. Storage Stability of the Spray-Dried Oil Powders

To evaluate the storage stability of the spray-dried oil powders, the newly prepared oil powders were sealed in aluminum laminated polyethylene (ALPE) pouches. The pouches were placed in a constant temperature and humidity incubator at storage conditions of 30 °C and 60% relative humidity (RH). Oil powders were withdrawn at different time intervals (30, 60, 90, 120, 150, and 180 days, respectively) for analyses, and the moisture content, surface oil content, total oil content, acidity value, and peroxide value were measured as described in the preceding text.

### 2.8. Animal Experiment

Thirty-two male C57BL/6J mice at 4–5 weeks old (22–23 g) were split at random into four groups (*n* = 8): NC group, DSS group, FS group, and FSP group. Mice in the NC group (normal control group) were administered with 0.2 mL/day of sterile water for 21 days. Mice in the DSS group (colitis model group) were administered DSS (2%) drinking water for seven days, followed by administration with distilled water for 14 days. Mice in the FS group and FSP group received DSS (2%) oral solution for seven days and were then treated with flaxseed oil and dried flaxseed oil powders for 14 days, respectively. The flaxseed oil or flaxseed oil powder was dissolved in 0.1% flaxseed gum solution [17] for subsequent oral administration. The dose at 500 mg/kg·Bw/day that has high therapeutic efficacy in colitis was referred to in our preliminary experiments. This animal experiment was approved by the Animal Ethics Committee of Huazhong Agricultural University (Permission HZAUMO-2021-0170).

Colitis-related parameters, including body weight loss and colon length, were measured after the mice were sacrificed. Haematoxylin and eosin (HE) stain was carried out for the distal colon tissues fixed in 4% paraformaldehyde solution, and microscopic observation at a magnification of 40X was performed for histologic assessment. Serum cytokines levels (IL-1β, IL-6, TNF-α, and IL-10) were detected by ELISA (elabscience Biotechnology Co., Ltd., Wuhan, China) following the supplier’s recommendations. Fecal DNA was extracted using the DNA Stool Mini Kit and sequenced using the Illumina Miseq platform (2 × 150 pair-end) by Shanghai Paiseno Biological Technology Co., LTD. Bioinformatic analysis of the microbial component and structure in gut microbiota was implemented according to Yang et al. [18].

### 2.9. Statistical Analysis

The experiments were conducted in triplicate. The data are reported as means ± standard deviations. Difference significance in the gut microbiota across subgroups was processed using One-way ANOVA. *p*-value < 0.05 was set as significant.

## 3. Results and Discussion

### 3.1. Effect of Raw Material Compositions on Spray-Dried Flaxseed Oil Powders

During the encapsulation of oil with a spray drying method, various factors such as raw material compositions, emulsification conditions, and spray drying conditions can affect the properties of the powered oil products. Four different factors, including wall ratio, core/wall ratio, solid concentration, and emulsifier content, were optimized using the orthogonal *L_16_ (4) ^4^* experiments. A total of 16 experimental trials were spray dried, and the encapsulation efficiency and encapsulation yield results were determined as indicators.

**Figure 1:**
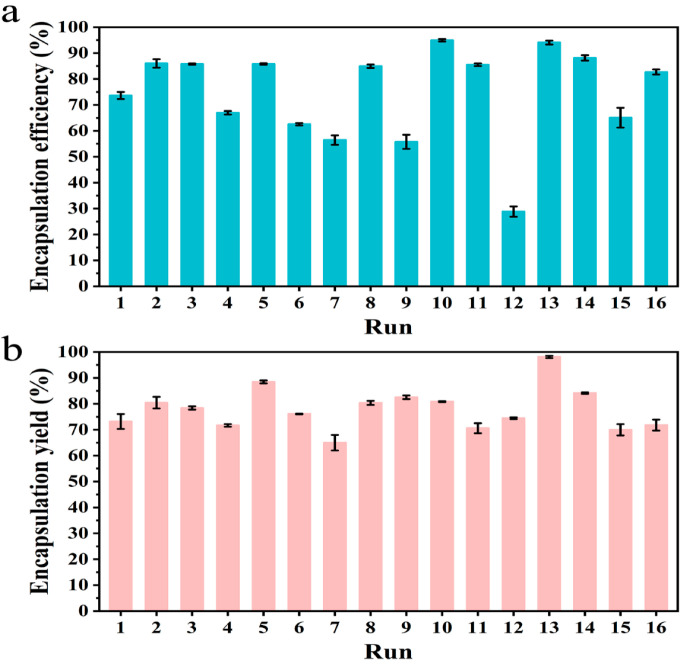
The encapsulation efficiencies (**a**) and encapsulation yields (**b**) of the flaxseed oil powders from the orthogonal *L16 (4) ^4^* experiments.

The encapsulation efficiency results are shown in Figure 1a, with a range from 28.8% to 94.95%. There were two experiments that showed encapsulation efficiency higher than 90%; they were run 10 (94.95%) and run 13 (94.09%), respectively. The corresponding analyses are exhibited in Table S2, showing that the optimal levels of raw material compositions are A4 (wall ratio, 1:0.2), B2 (core/wall ratio, 1:0.9), C3 (solid concentration, 42%), and D2 (emulsifier content, 1%). The encapsulation yields of these experiments were then evaluated (Figure 1b, Table S3), which showed that the optimal level of raw material compositions was A4 (wall ratio, 1:0.2), B1 (core/wall ratio, 1:1), C3 (solid concentration, 42%), and D2 (emulsifier content, 1%). In addition, only the result of run 13 exceeded 90% (Figure 1b). Combining these results and analyses, the raw material compositions at wall ratio (1:0.2), core/wall ratio (1:1), solid concentration (50%), and emulsifier content (1%) were selected for further experiments. The total oil and surface oil contents of the oil microcapsules under this condition were 49.05% and 2.90%, respectively. The encapsulation efficiency is comparable to other spray-dried flaxseed oil microcapsules which have similar or lower oil loading. Farzaneh Mohseni encapsulated flaxseed oil using the combination of oxidized tannic acid-gelatin and flaxseed mucilage and obtained microcapsules with 50% oil loading and 94.2% encapsulation efficiency, which are higher than that obtained in a similar work conducted by Pratibha Kaushik [19,20]. 

### 3.2. Effect of Emulsification and Spray Drying Conditions on Spray-Dried Flaxseed Oil Powders

Based on the results of these experiments, the effects of emulsification and spray drying conditions on the characteristics of oil powders were then investigated. Homogenization pressure, homogenization cycles, and inlet air temperature were evaluated in this section, and the surface oil and total oil contents were selected as the key indicators. To optimize every single factor, a single variable was determined in the given scope: the homogenization pressures were tested at 20, 40, 60, and 80 MPa, the homogenization cycles at one, two, three, and four times, the inlet air temperature at 150, 160, 170, and 180 °C, and the emulsion feeding rate at 4, 6, 8, and 10 L/h. The raw material compositions were the same as the optimal determined in the preceding text.

During the emulsion preparation process, when the homogenization pressures increased from 20 to 40 MPa, the surface oil contents of the oil powders decreased significantly from 3.7% to 2.94%. However, with an excessive increment of this condition from 40 to 80 MPa, the surface oil contents of the oil powders barely changed. Another parameter that can affect the surface contents during the emulsion process is the homogenization cycle, and the surface oil contents will gradually be decreased from 3.12% to 2.18% when this factor increases from one time to four times. The results showed that moderate homogenization conditions could modulate the surface oil contents of spray-dried oil powders, and further increments of both homogenization pressures and cycles may cause no changes to surface oil contents. These phenomena were also observed in studies conducted by Quoc Dat Lai and Rudra Pangeni, in which they reported that moderate pressures and cycles could decrease the droplet size of emulsion and then affect the encapsulation efficiency or emulsion stability, while higher pressures and more cycles may not change these characteristics and even decrease them [21,22]. 

When the inlet air temperature was increased from 150 to 180 °C, the surface oil content decreased significantly from 2.78 to 1.07, and the inlet air temperature at 170 °C resulted in the lowest surface oil content. This might be due to the accelerated drying rate under higher inlet air temperature, promoting the formation of powder shells which can limit the leaching of oil from the powders. Similar behavior was reported in a study conducted by Mortaza Aghbashlo [23]. In addition, if the emulsion feeding rate was too high (10 L/h), the surface oil content was higher than 1.02%, while with a lower feeding rate at 4, 6, and 8 L/h, the surface oil content was significantly lower (~0.2%). Similar discoveries were reported by Phu Thuong Nhan Nguyen in the encapsulation of essential oil, and the increased surface oil contents under a higher feeding rate could be explained by larger droplets during the encapsulation process [24].

Therefore, through sequential experiments to determine the raw material compositions, emulsification conditions, and spray drying conditions, the optimal conditions for the preparation of flaxseed oil powders were obtained (Table 3). The conditions were wall ratio (1:0.2), core/wall ratio (1:1), solid concentration (50%), emulsifier content (1%), homogenization pressure (40 Mpa), homogenization cycles (three times), and inlet air temperature (170 °C). The availability of encapsulating a different oil type (safflower seed oil) in optimal conditions was further investigated, and the physicochemical properties of these powders were studied and compared.

Through a series of experiments to optimize the optimal conditions for oil powders, an encapsulation system that has high encapsulation efficiency and yield was obtained. The possibility of encapsulating different plant-based oil types (flaxseed oil and safflower seed oil) and the properties of the obtained oil powders were evaluated.

### 3.3. Physicochemical Properties of Oil Emulsions and Powders

#### 3.3.1. Emulsion Droplet Size

**Figure 2:**
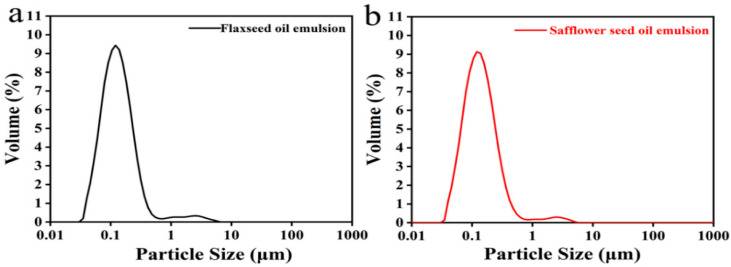
Particle size distributions of flaxseed oil emulsion (**a**) and safflower seed oil emulsion (**b**).

Figure 2 shows the emulsion droplet sizes of two emulsions from different oil types: flaxseed oil emulsion and safflower seed oil emulsion. The particle size distributions of both emulsions were comparable and displayed largely unimodal distributions. The surface-weighted mean diameter (D [3,2]) and volume-weighted mean diameter (D [4,3]) of flaxseed oil and safflower seed oil emulsions are 0.130 and 0.211 μm, and 0.123 and 0.190 μm, respectively. The emulsion droplet sizes are significantly smaller than the oil emulsions fabricated from similar wall materials [25,26]. The emulsion droplet size results in Table 4 were almost independent of the oil types, suggesting that the optimal wall material combinations and emulsification conditions were available to prepare stable feed emulsions necessary for microencapsulation by spray drying that contains a high oil loading content of up to 50% *w*/*w* oil in the final powder products.

#### 3.3.2. Powder Morphology

**Figure 3:**
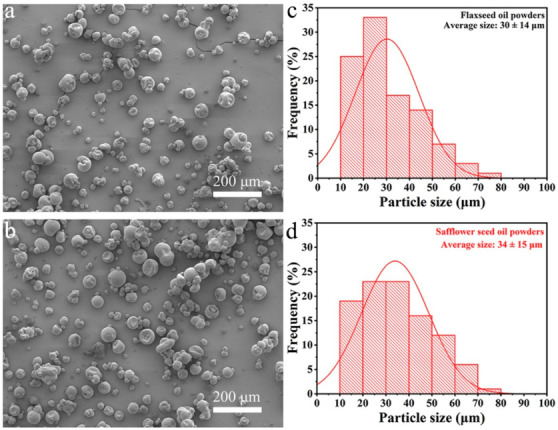
SEM images and corresponding particle size distributions of flaxseed oil powders (**a**,**c**) and safflower seed oil powders (**b**,**d**).

The microstructures of the oil powders containing flaxseed oil and safflower seed oil were observed by SEM. As shown in Figure 3a,b, both particles displayed spherical shapes with smooth surfaces, and little cracks or shrinkages were observed, suggesting that these wall material combinations are capable of retaining the core oil within the microcapsules for protection. The wrinkled surface in some powders can be attributed to the high inlet temperature that caused rapid shrinkage of sprayed emulsion droplets, which is inevitable during the spray drying process. Similar phenomena were also observed in previous research [27,28]. The mean diameter and size distribution of the particles were further determined by analyzing these images with ImageJ software (Figure 3c,d). The particle mean diameters of flaxseed oil and safflower seed oil powders were 30 ± 14 μm and 34 ± 15 μm, respectively, and these powders exhibited almost uniform size distributions ranging from 10 to 70 μm and 13 to 73 μm, respectively. These results demonstrate that encapsulating different oil samples using an encapsulation system has little influence on the microstructures and size distributions of the final products. 

#### 3.3.3. Physicochemical Properties of the Oil Powders

Table 5 shows some physicochemical characteristics of oil powders containing flaxseed oil and safflower seed oil prepared through optimal conditions. The moisture contents of flaxseed oil and safflower seed oil powders were 1.31% and 1.43%, respectively, lower than the recommended level of 4~5% for spray-dried powders used for food application [29,30]. Previous studies demonstrated that the high moisture content of powders can cause agglomeration, microbial growth, and accelerated deterioration, which are undesirable for the long-term storage of spray-dried powders [31]. A significantly low level of moisture content would minimize the risks and be beneficial to the storage of the powders.

The total oil contents were similar for these two kinds of oil powders and close to the theoretical value, with high total oil contents of ~50%. It is challenging to achieve such high oil loading as the increment of oil contents can result in increasing surface oil content, which is an important characteristic of oil powder. The presence of oil on the surface of the powders will make these powders susceptible to oxidation and cause deterioration of the quality of the powders. In this study, the surface oil contents of the flaxseed oil and safflower seed oil powders were only ~0.20%, which is significantly lower than some other spray-dried oil powders with the same level of oil loading. Through these two statistics, the encapsulation efficiencies can be obtained, which represent the proportion of oil that is surrounded by the wall materials and less exposed to the outer environment. Owing to the significantly low surface oil contents, the encapsulation efficiencies were obviously higher (~99.6%) than the other spray-dried oil powders at the same level of oil loading [32]. 

As the preparation of oil powders must go through a series of processing processes, the acidity values and peroxide values of the final powder products can be affected during this period. Compared with the initial oil, the encapsulated oil powders were more oxidized. The initial acidity values of flaxseed oil and safflower seed oil were 0.56 ± 0.02 and 0.48 ± 0.03 mg/g, and their initial peroxide values were 0.05 ± 0.03 and 0.06 ± 0.02 g/100 g, respectively. After emulsion preparation and spray drying processes, the acidity values of both powders increased slightly to 0.60 ± 0.04 and 0.52 ± 0.03 mg/g, and the peroxide values increased to 0.09 ± 0.01 and 0.08 ± 0.02 g/100 g, respectively. The peroxide values, which show the oxidation levels of oil before and after encapsulation, increased during this period, and this is due to the homogenization and high-temperature treatments during the encapsulation process. These results are in agreement with the observations of some similar studies, which obtained encapsulated oil samples with peroxide values ranging from 0.08 to 0.11 g/100 g (unit conversion from meq/kg oil) after spray drying treatments [5,33]. 

### 3.4. Storage Stability

**Figure 4:**
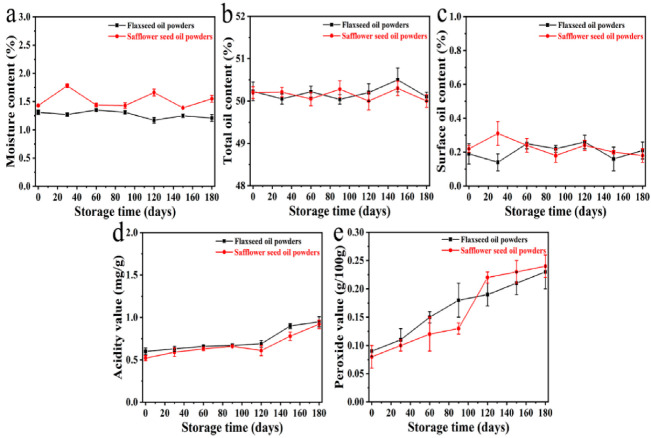
Moisture contents (**a**), total oil contents (**b**), surface oil contents (**c**), acidity values (**d**), and peroxide values (**e**) of the flaxseed oil powders and safflower seed oil powders over a storage period of 180 days.

The storage stability of the spray-dried powders was assessed to optimize the raw material compositions and operating conditions. Figure 4 shows some of the physicochemical properties of the flaxseed oil powders and safflower seed oil powders placed in 30 °C and 60% RH conditions over a storage period of 180 days. These powders had high stability in moisture, total oil, and surface oil contents, as the parameters of both oil powders were barely changed during this storage period. Though acidity values and peroxide values of both powders were gradually increased from 0 to 180 days, the acidity values were less than 1 mg/g, and peroxide values were less than 0.25 g/100 g. Compared with studies that had similar initial peroxide values after encapsulation, the initial oxidation levels did not guarantee oil powders with similar quality after long-period storage. The peroxide values increased from 0.08 to 0.64 g/100 g after 45 days of storage at 25 °C and increased from 0.02 to 0.19 g/100 g after 30 days of storage at room temperature. Despite the lower storage temperature in these studies, the peroxide values were significantly higher than those observed during the same storage time in our work [33,34]. These results suggest that the proposed encapsulate system has high efficiency in maintaining the stability of flaxseed oil and safflower seed oil over a long period.

### 3.5. Alleviation of DSS-Induced Colitis

**Figure 5:**
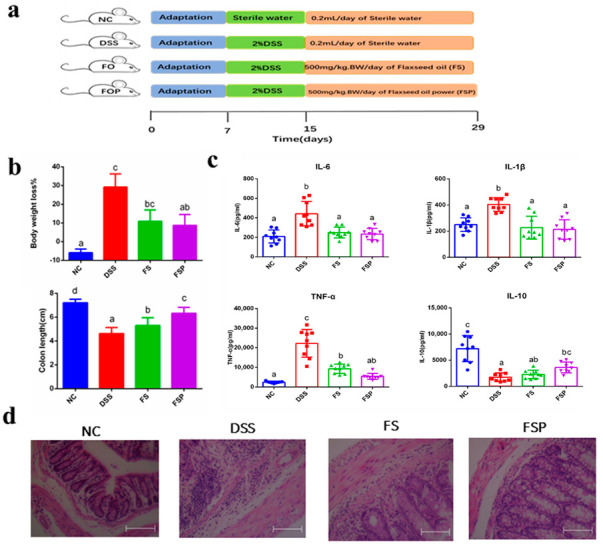
The schematic representation of the experiment (**a**), mice body weight change and colon length (**b**), inflammatory cytokine (IL-6, IL-1β, TNF-α and IL-10) levels in colonic tissue (**c**) and histopathological changes and assessment of colonic tissues from the experiment group (**d**). Groups are labeled from smallest to largest in the order of mean value. Mean values with different letters are significantly different (*p* < 0.05).

An in vivo animal experiment focusing on the effect of oral treatment with the flaxseed oil powder on colitis induced by DSS was performed (Figure 5a). A progressive increase in body weight of the mice in the NC group during the experiment (4.1% at the endpoint) was observed, while DSS mice revealed significant body weight loss (−28.3%) at the end of the experiment. Mice supplied with flaxseed oil had less body weight, and mice administrated with flaxseed oil power had the least body weight loss when compared to DSS-treated colitis mice (Figure 5b). In addition, the colon length of DSS mice was shorter than that of NC mice, and both FS and FSP mice showed improved colon length compared to DSS mice (Figure 5b). Colon tissues in DSS mice exhibited a loss of goblet cells and epithelial cells, severe mucosal epithelial damage, and deformed crypt glands. After the flaxseed oil intervention, there was more complete mucosal structure than in the DSS mice group, particularly in the FSP group (Figure 5d). Several studies have shown that flaxseed oil rich in n-3 PUFA relieved colitis induced by DSS in rats through regulation of the oxidative stress, inflammatory response and intestinal microbiota [35], adjustment of the normal expression of the inflammatory eicosanoids and cytokines including interleukin-6 (IL-6) and NF-κB p65 subunit [36], and suppression of endotoxin-triggered inflammation by blocking the TLR4/MyD88/NF-κB pathway in the liver [37]. These results suggest that treatment with flaxseed oil alleviates DSS-induced colitis symptoms in line with previous reports. It is important to note that the effect of spray-dried flaxseed oil powder was superior to normal liquid flaxseed oil.

IBD is characterized by immune dysfunction, imbalanced cytokine network, and mucosal-associated inflammatory progression [38]. Patients with IBD showed elevated levels of pro-inflammatory cytokines in the intestinal mucosa, such as TNF-α, IL-1β, and IL-6 [39]. Therefore, the serum pro-inflammatory cytokines (IL-1β, TNF-α, IL-6) and anti-inflammatory cytokine (IL-10) levels in the tested mice were determined. As shown in Figure 5c, DSS treatment significantly increased serum TNF-α (*p* < 0.01), IL-1β (*p* < 0.05), and IL-6 (*p* < 0.05) levels compared to controls. The contents of these cytokines were significantly reversed in FS mice and FSP mice. When compared with the mice in the NC group, the cytokine IL-10 level in the colon tissue of the DSS group was significantly reduced (*p* < 0.01). However, compared with the DSS group, only the mice supplemented with flaxseed oil powder (FSP Group) showed an increase in IL-10 levels (*p* < 0.05). Researchers found that n-3 PUFAs could protect mice from intestinal inflammation via downregulating the TLR/NOD pathway [40], in which the latter leads to modulating pro-inflammatory cytokines, such as IL-1β and IL-18 [41]. Our results indicate that flaxseed oil could inhibit the release of cytokines, possibly by suppressing inflammasome activation and maturation and production of IL-1β, just like other oils rich in n-3 PUFA. Moreover, spray drying did not affect the ability of flaxseed oil to regulate inflammatory cytokines and even seemed to increase it.

### 3.6. Regulation of Gut Microbiota

**Figure 6:**
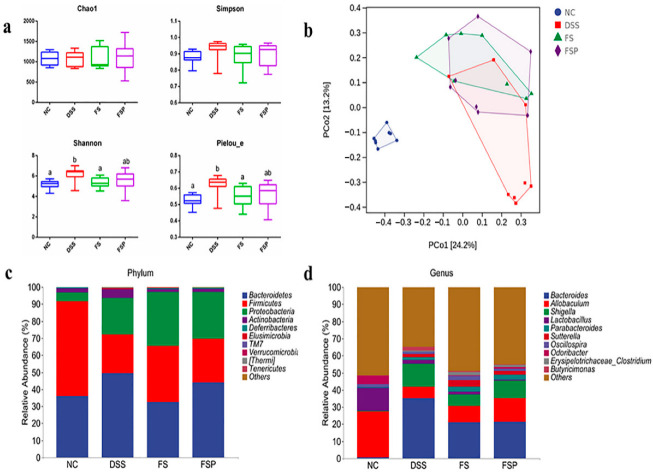
The alpha diversity (Chao1, Shannon, Simpson, and Pielou_e indexes) in different groups (**a**), PCoA plot of unweighted UniFrac distances of beta diversity (**b**), and community bar plots of phylum and genus level (**c**,**d**). Groups are labeled from smallest to largest in the order of mean value. Mean values with different letters are significantly different (*p* < 0.05).

16S rDNA sequencing technology was used to analyze the microbiota community variations of experimental mice. In terms of α-diversity indexes, Chao1 represents the community richness of gut microbiota, and Simpson, Shannon, and Pielou_e indexes indicate the community diversity. Figure 6a shows that the Shannon and Pielou_e indexes of the DSS group were higher than those of the NC group (*p* < 0.05). After treatment with flaxseed oil, the Chao1 index was relieved. Additionally, no significant difference in the Simpson index of gut microbiota was observed among the experimental groups. As shown in Figure 6b, principal coordinate analysis showed a distinct cluster of mice from the NC and DSS groups. The administration of flaxseed oil and flaxseed oil powder can change the structure of the intestinal flora induced by DSS treatment. The composition of the bacterial community at the phylum and genus levels was identified (Figure 6c,d). At the phylum level, the dominant taxa of the gut microbiota in each group were *Firmicutes*, *Bacteroidetes*, and *Proteobacteria*, accounting for ~95.5–99.8%. As many reports concluded, *Firmicutes* and *Bacteroidetes* are the absolute dominant phylum in both human and animal intestinal communities [42,43], and these two main communities are associated with host energy metabolism homeostasis [44]. In this study, *Firmicutes* and *Bacteroidetes* occupied dominance in the gut microbiota was not changed in the DSS-induced mice, which led us to pay more attention to the changes at genus levels or at other phyla. This result was consistent with our previous studies [17,45] and other research work [46]. 

At the genus level, the dominant bacterial genera detected in the tested group were different. *Allobaculum*, *Lactobacillus*, and *Odoribacter* were the top three taxa in the NC group, whereas *Bacteroides*, *Allobaculum*, and *Shigella* were the top three taxa in DSS, FS, and FSP groups. In addition, Figure 6d shows that the abundance of *Allobaculum* was significantly lower in the DSS group than in the NC group (*p* < 0.05), while supplementation with flaxseed oil powder (FSP group) but not flaxseed oil (FS group) increased the proportion of *Allobaculum* in DSS-induced mice. Additionally, in the FSP group, the relative abundance of *Bacteroides* significantly declined compared with the DSS group (*p* < 0.05). *Allobaculum* is considered a beneficial bacteria since it could inhibit mice weight gain by interfering with energy metabolism [47]. As a potential human intestinal mucin degrader, it is also reported to protect intestinal barrier function by producing short-chain fatty acids (SCFAs) [48]. On the other hand, pathogenic bacteria such as *Bacteroides* are reported to be negatively related to some special SCFAs [49]. Here, in the mice supplemented with flaxseed oil powder but not flaxseed oil, the drop of beneficial *Allobaculum* promoted the reparation of the broken intestinal barrier induced by DSS and inhibited the proliferation of harmful genera such as *Bacteroides* via the production of SCFAs. 

**Figure 7:**
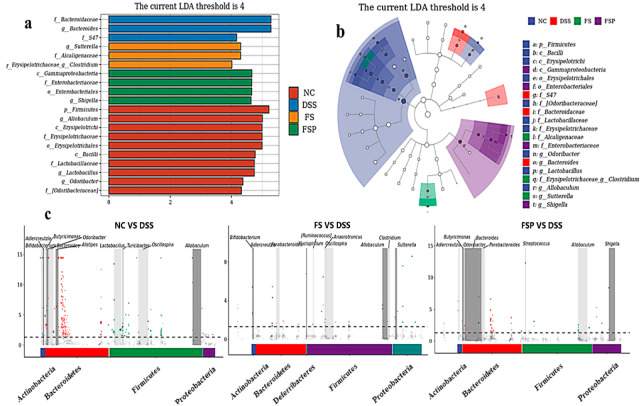
Taxonomic histogram (**a**) and cladogram (**b**) generated by LEfSe analysis and matastats analysis (**c**).

LDA-LefSe was used to analyze the prominent taxa in each group and reveal changes in gut microbiota composition. According to Figure 7a,b, it is possible to observe that *Allobaculum*, *Lactobacillus*, and *Odoribacter* were enriched in the NC group, and *Bacteroides* were enriched in the DSS group. *Clostridium* and *Shigella* were enriched in the FS and FSP groups, respectively. Matastats were carried out to identify the significantly different taxa between each two group pairs (Figure 7c). Mice supplemented with flaxseed oil promoted more *Ruminococcus* and *Anaerotruncus* from *Firmicutes* phylum and less *Bacteroides* from *Bacteroidetes* phylum compared to untreated DSS-colitis mice. In addition to this effect, consumption of flaxseed oil powder induced more *Allobaculum* and *Odoribacter* after DSS treatment. These results matched the changed genus shown in Figure 6c,d, which suggests that FSP impacts the intestinal microbiome at different taxonomic levels by improving the proportion of SCFAs producers, *Allobaculum* and *Odoribacter*, and inhibiting the growth of bacterial species associated with diseases and inflammation such as *Bacteroides and Shigella*.

Intestinal dysbiosis induced by inflammatory bowel disease (IBD) was associated with intestinal barrier function via excessive inflammatory response [50]. Considering the fact that many bacterial species are closely associated with human diseases, the inner mucus layer was proved an important niche [51]. As discussed before, *Allobaculum* was known to be related to the host epithelium, which could maintain intestinal barrier function by secreting acetic acid [18] and 5-HT [52]. Here, both flaxseed oil and flaxseed oil powder displayed a positive regulatory effect on the bacterial community in colitis mice. Mice that accepted the flaxseed oil powder treatment had a remarkably increased abundance of *Allobaculum* and decreased proportion of infectious diseases associated the *Shigella* genus, which might improve the integrity and function of the intestinal barrier in colitis mice. However, the deeper mechanism between these regulated genera and the improvement of the intestinal barrier by the plant oil powder consumption needs to be further investigated.

## 4. Conclusions

This study systematically investigated the preparation of spray-dried plant-based oil powders that contain an oil loading of 50%. The optimal conditions, including raw material compositions, emulsification conditions, and spray drying conditions, were determined through designing a series of orthogonal experiments and single-factor experiments. The surface oil content was lower than 0.19%, and the encapsulation efficiency was significantly high (99%). The oil powders exhibited long-term stability under 30 °C and 60% relative humidity. The encapsulated flaxseed oil powders were shown to alleviate a series of DSS-caused inflammatory symptoms and modulate the intestinal microbiota in mouse models. Notably, flaxseed oil powder exhibited enhanced anti-colitis activity by improving the physiological indications and gut dysbiosis compared with lipid flaxseed oil.

## Figures and Tables

**Table 1:** Selected raw material factors and respective levels.

Level	Factors			
	Wall Ratio	Core/Wall Ratio	Solid Concentration	Emulsifier Content
1	0.6:1	1:1	26%	0.5%
2	1:1	1:0.9	34%	1%
3	1:0.6	1:0.8	42%	2%
4	1:0.2	1:0.7	50%	5%

**Table 2:** Design of orthogonal *L_16_ (4) ^4^* experiments.

Run	Factors			
	Wall Ratio	Core/Wall Ratio	Solid Concentration	Emulsifier Content
1	0.6:1	1:1	26%	0.5%
2	0.6:1	1:0.9	34%	1%
3	0.6:1	1:0.8	42%	2%
4	0.6:1	1:0.7	50%	5%
5	1:1	1:1	34%	2%
6	1:1	1:0.9	26%	5%
7	1:1	1:0.8	50%	0.5%
8	1:1	1:0.7	42%	1%
9	1:0.6	1:1	42%	5%
10	1:0.6	1:0.9	50%	2%
11	1:0.6	1:0.8	26%	1%
12	1:0.6	1:0.7	34%	0.5%
13	1:0.2	1:1	50%	1%
14	1:0.2	1:0.9	42%	0.5%
15	1:0.2	1:0.8	34%	5%
16	1:0.2	1:0.7	26%	2%

**Table 3:** Effects of homogenization pressure, homogenization times, inlet air temperature, and emulsion feeding rate on surface oil contents of flaxseed oil powders.

Factors	Level	Surface Oil (%)
Homogenization pressure	20 MPa	3.70 ± 0.12
	40 MPa	2.94 ± 0.14
	60 MPa	2.88 ± 0.09
	80 MPa	2.90 ± 0.11
Homogenization cycles	1 time	3.12 ± 0.13
	2 times	2.90 ± 0.09
	3 times	2.22 ± 0.10
	4 times	2.18 ± 0.04
Inlet air temperature	150 °C	2.78 ± 0.22
	160 °C	2.22 ± 0.15
	170 °C	1.02 ± 0.07
	180 °C	1.07 ± 0.13
Emulsion feeding rate	4 L/h	0.22 ± 0.03
	6 L/h	0.23 ± 0.01
	8 L/h	0.19 ± 0.06
	10 L/h	1.02 ± 0.02

**Table 4:** Particle size results of flaxseed oil and safflower seed oil emulsions.

	Flaxseed Oil Emulsion	Safflower Seed Oil Emulsion
D [0.1]	0.067 μm	0.069 μm
D [0.5]	0.135 μm	0.140 μm
D [0.9]	0.301 μm	0.321 μm
D [3,2]	0.116 μm	0.120 μm
D [4,3]	0.234 μm	0.226 μm
Span	1.732	1.797

**Table 5:** Physicochemical properties of the flaxseed oil and safflower seed oil powders.

Parameters	Flaxseed Oil Powders	Safflower Seed Oil Powders
Moisture content (%)	1.31 ± 0.04	1.43 ± 0.01
Total oil (%)	50.23 ± 0.22	50.20 ± 0.14
Surface oil (%)	0.19 ± 0.06	0.22 ± 0.02
Encapsulation efficiency (%)	99.6	99.6
Acidity value (mg/g)	0.60 ± 0.04	0.52 ± 0.03
Peroxide value (g/100 g)	0.09 ± 0.01	0.08 ± 0.02

## Data Availability

The data supporting the findings of this study are available within the article.

## Supplementary Materials

The following supporting information can be downloaded at: https://www.mdpi.com/article/10.3390/foods11192993/s1, Table S1: Fatty acid compositions of flaxseed oil and safflower seed oil; Table S2 and S3: Evaluation indices of the encapsulation efficiency and encapsulation yield of the orthogonal *L_16_ (4) ^4^* experiments.
