# Supplementary material for: Encapsulation of Functional Plant Oil by Spray Drying: Physicochemical Characterization and Enhanced Anti-Colitis Activity
Source: Foods. 2022 Sep 26;11(19):2993. doi: 10.3390/foods11192993 (PMC9562653; doi:10.3390/foods11192993)
Supplement: Supplementary file 1 [file foods-11-02993-s001.zip › foods-1870617-supplementary.pdf]

Supplementary materials

Table S1. Fatty acid compositions of flaxseed oil and safflower seed oil

**Fatty acid composition**		**Flaxseed oil**	**Safflower seed oil**
Poly unsaturated fatty acids	linolenic acid	56.6%	-
	linoleic acid	15.3%	78%
Mono unsaturated fatty acids	oleic acid	18.2%	10.9%
Saturated fatty acids		9.8%	11%

Table S2. Evaluation indices of the encapsulation efficiency of the orthogonal L_{16} $(4)^4$ experiments.

	Wall ratio (A)	Core/wall ratio (B)	Solid concentration (C)	Emulsifier content (D)
K1	312.37	309.24	304.38	247.00
K2	289.68	331.63	265.67	350.55
K3	265.05	292.76	314.61	349.19
K4	329.98	263.45	312.42	250.34
K1 avg	78.09	77.31	76.09	61.75
K2 avg	72.42	82.91	66.42	87.64
K3 avg	66.26	73.19	78.65	87.30
K4 avg	82.49	65.86	78.10	62.58
R	16.23	17.05	12.23	24.71
Optimal level	A4	B2	C3	D2
Order of importance		D > B > A >C		

Table S3. Evaluation indices of the encapsulation yield of the orthogonal L_{16} $(4)^4$ experiments.

	Wall ratio (A)	Core/wall ratio (B)	Solid concentration (C)	Emulsifier content (D)
K1	303.68	342.29	291.62	296.72
K2	309.88	321.51	313.29	329.47
K3	308.35	283.89	325.44	319.46
K4	323.99	315.56	315.56	300.25
K1 avg	75.92	85.57	72.90	74.18
K2 avg	77.47	80.38	78.32	82.37
K3 avg	77.09	70.97	81.36	79.86
K4 avg	81.00	78.89	78.89	75.06
R	5.08	14.60	8.45	8.19
Optimal level	A4	B1	C3	D2
Order of importance		D > C > A >B		
